# Virtual Planning, Control, and Machining for a Modular-Based Automated Factory Operation in an Augmented Reality Environment

**DOI:** 10.1038/srep27380

**Published:** 2016-06-07

**Authors:** Yun Suen Pai, Hwa Jen Yap, Siti Zawiah Md Dawal, S. Ramesh, Sin Ye Phoon

**Affiliations:** 1 Graduate School of Media Design Keio University, 4-1-1, Hiyoshi, Kohoku, Yokohama, 223-8526, Japan.; 2 Department of Mechanical Engineering, Faculty of Engineering, University of Malaya, 50603, Kuala Lumpur, Malaysia

## Abstract

This study presents a modular-based implementation of augmented reality to provide an immersive experience in learning or teaching the planning phase, control system, and machining parameters of a fully automated work cell. The architecture of the system consists of three code modules that can operate independently or combined to create a complete system that is able to guide engineers from the layout planning phase to the prototyping of the final product. The layout planning module determines the best possible arrangement in a layout for the placement of various machines, in this case a conveyor belt for transportation, a robot arm for pick-and-place operations, and a computer numerical control milling machine to generate the final prototype. The robotic arm module simulates the pick-and-place operation offline from the conveyor belt to a computer numerical control (CNC) machine utilising collision detection and inverse kinematics. Finally, the CNC module performs virtual machining based on the Uniform Space Decomposition method and axis aligned bounding box collision detection. The conducted case study revealed that given the situation, a semi-circle shaped arrangement is desirable, whereas the pick-and-place system and the final generated G-code produced the highest deviation of 3.83 mm and 5.8 mm respectively.

An effective simulation is one that is able to place a user in a situation which is close, if not completely identical to the scenario of which the system is attempting to simulate. The correct term for providing a user with a sense of presence, or “being there”, is immersive, where in the world of virtual interaction, is defined as a complex technology that replaces real-world sensory information with synthetic stimuli such as 3D visual imagery, spatialised sound, and force or tactile feedback^1^. In manufacturing, the advent of computer numerical control (CNC) machining creates a form of ubiquitous computing, and provides an effective simulation as it becomes a necessity. CNC simulations have been developed in virtual environments for numerically controlled (NC) tool path verification and machining process optimisation^2^. However, limitations still exist even though virtual reality (VR)-based systems have already been applied broadly in the manufacturing industry. Firstly, the system is usually costly, requires powerful hardware, and separates the simulation aspect from the machining aspect, meaning the user has to adjust the experience gathered from the 3D graphic environment to the real machining environment^3^. Secondly, the system is so tightly integrated that it is difficult to support continuous improvement and lessens flexibility, considering that a fully autonomous CNC manufacturing environment from start to finish involves many steps, not just the machining aspect^4^. For example, how does one determine the placement of machines to accommodate the CNC machining aspect, and how does one place the stock material onto the worktable in an autonomous system?

This brings forward the demand for a new technology, dubbed augmented reality (AR). AR is a rapidly growing field of research that aims to fully integrate virtual with real environment. AR has been developed since the early 90s, but has only recently been emerging as one of the forefront of technology, mainly due to the rise of popularity in smartphones and tablets^5^. Some of the systems that have been developed for production process are related to layout planning^6^,^7^,^8^,^9^,^10^, product design^11^,^12^,^13^,^14^,^15^, assembly^16^,^17^,^18^,^19^,^20^, robot programming^21^,^22^,^23^,^24^,^25^,^26^, and autonomous machining^3^,^27^,^28^,^29^. This proves that AR can be applied in many fields of research and even in consumer products due to the lower system requirement being one of the main contributing factors. By enhancing the users’ understanding and interaction with the manufacturing environment, shorter lead time and lower manufacturing costs can be achieved^30^. In addition, providing simulation in a real environment partly removes the time-consuming geometric and kinematic modelling of the machine tools and accessories in the real environment, which helps to improve the simulation efficiency.

## Related Studies

The field of visualisation is stepping into a new era of development, with emerging hardware support by large corporations due to the realisation of a promising future associated with this technology. It has been applied in mobile robotics, entertainment industries, and even injury treatment^31^,^32^, where each of them is derived from enhanced computer graphics that often uses multi-agent systems^33^. This is due to the degree of immersion it provides, which is a key factor in any form of simulation. A successful simulation is when the system is able to fully immerse the user in its surroundings for a better understanding. Immersion comes in many ways, and visualisation, as the name implies, improves that through sight. In the manufacturing industry, the use of robotic arms or computer-controlled machines always require a form of programming to teach them the actions that need to be taken depending on the situation. If this task is improved or enhanced visually^34^,^35^, then it is safe to say that simulating the automated system will yield higher productivity due to higher immersion and intuitiveness. This section will discuss the past and present developments of the application of virtual or augmented reality in layout planning, robot programming, and machining, to determine the existing gaps.

## Layout Planning

The design of a facility’s layout is associated with the allocation of machines, work cells, and departments which play a role in ensuring an efficient and effective operation^36^. Many researches were conducted to develop simulation models in the manufacturing system design, as engineers need to reduce any uncertainties present, such as assessment errors which are hard to determine in traditional facility layouts. A recent survey was conducted to establish the problems in today’s layout planning process and it was found that graphical tools are able to create a more efficient and attractive environment that can replace existing planning processes^37^. Since flexible manufacturing system (FMS) is a system that integrates its elements tightly, the relation between them is often hard to compute^38^. Therefore, there is a demand for an analysis method that avoids any substantial loss in labour time, money, and resources. AR was used to aid the planning process of manufacturing systems with the key advantage of modelling 3D objects in the actual factory. By using AR as a form of user interface, any user will be able to freely manipulate the overall layout design on a table-top, which is extremely user-friendly^39^.

A 2D view of a system is often not easy to understand and evaluate. Therefore, virtual systems offer a depth perspective that is not possible for a 2D view to provide, while at the same time it ensures the ability to re-layout existing factory layouts^6^. Moreover, it is worthwhile to mention that an AR system can achieve these benefits without any additional computing cost of a VR system. At this moment, AR technology has found a place in collaborative design work, maintenance, assembly, robot path planning, CNC simulation, and of course, plant layout planning^30^. Specifically, it was dubbed as AR-based factory layout planning (FLP) systems where it allows users to lay out virtual objects to integrate human intuitiveness with layout design process.

## Industrial Robots

Kinematic modelling of an industrial robot is a vital part of this study as the main aim of this module is to manipulate a virtual robotic arm effectively in an AR environment. The Denavit-Hartenberg-based (D-H) method is the most popular approach for kinematic analysis^40^ and is used for the robotic arm manipulator in this study. The mathematics and notations of the robot’s forward kinematics can be best determined through the D-H method regardless of its sequence or complexity, and the location of the joint is dependent on the previous joint’s location which can be calculated using transformation matrices. Integration between layout planning and robot programming was achieved in the past with VR, where a modular system was developed that was able to pick and place objects that was planned earlier with the VR-layout system^9^. The system was similar in a sense that it was modular and encourages offline robot programming. However, a VR system is substantially different from AR in terms of execution, and although it has proven to be efficient, the system requires a higher computational capability and it does not cover the end product of what was being manipulated in the work cell. For example, the application and advantages of utilising AR in the field of industrial robot programming was discussed while presenting a novel approach towards planning a collision-free path in an unprepared environment^21^. The end-effector is always the main concern for robot programming; therefore, a Collision-Free Volume (CFV) was generated as a form of constraint to the position of the end-effector and to avoid collision with nearby objects.

## CNC Machining

Virtual CNC machining might be a new concept to some, but several studies have already analysed its possibility in application in industries, and some have already been applied. A study was made on the application of virtual machining in relation to its structural analysis as well as the challenges faced in the ongoing research in this field of technology^41^. The efficiency and capability of a machine tool highly depends on its structural dynamics, kinematics, CNC system, and the machining process itself. The technology we have today allows for the prediction of tool collision and path error checking by graphical means, but accuracy still remains a primary concern because a successful machining process is often the result of many more parameters that needs to be considered, such as chatter vibration. Existing virtual systems were reviewed in a recent article that covers from VR-based systems to mathematical modelling and NC-based simulation^42^. It was found that an unsolved issue until this very day is achieving flawless real-time simulation, even with the aid of web-based technologies. Much work is still required as human-computer interaction is a multi-disciplinary task with various approaches.

NC-based simulations have found a place among the research community for some time^43^, and image-space Boolean operations were even used to simulate cutting process in real-time since more than 20 years ago. A study then proved that shaded computer graphics were central in the design of solid models^44^. To achieve real-time shaded display, an extended Z buffer, or frame buffer structure was used. However, real-time changes in virtual models are still a challenge to overcome due to the low efficiency of Boolean operations in solid modelling. To further improve this, hardware inclusion like a 3D display is sorely needed, which also leads to the application of AR in CNC. The developed Augmented Reality Computer Numerical Control (ARCNC) system allows the operator to observe *in situ* simulation of the ball-end machining operation on a 3-axis vertical CNC machining centre, and the interaction between the real cutter and a virtual workpiece^3^. The system consists of the CNC machine, a camera, a display device, and a desktop PC for processing, where a physical simulation is performed with an enhanced dexel-based model.

Integrating AR in the rendering of a cutting simulation on a 3-axis CNC milling machine was also discussed where the cutting force was estimated based on a volumetric model-based material removal rate (MRR) and a fully interactive panel^29^. This means that the system architecture is divided into the AR-assisted CNC simulation and a monitoring station. The simulation portion renders either online or offline cutting simulation and the monitoring module executes the real machining process and monitors the information. The versatility of AR is further explored when it is used for validation purposes^45^. Since ARToolKit can be programmed to recognize and detect problematic cases in machining, as well as overlay the process information in real-time to the operator, it can be used to validate the NC-path of complex 5-axis milling machines.

CNC simulation is a form of automated machining, however, rarely does a system cover the steps and procedures taken before the machining is conducted. For example, the procedures involved include the methods of which the workpiece is placed on the worktable or the placement of machines in a work cell to facilitate a full production process. Therefore, the inclusion of a modular-based architecture greatly expands the scope of the system. For layout planning and industrial robot programming, both these systems can benefit greatly from AR. Planning a layout for machine placement can be difficult to visualise especially when factors such as the effects of the machine arrangements towards the production line, material travel time, and space required must be taken into account. With regards to robot programming, stopping a production line to programme a robot is extremely costly, and therefore a new form of offline programming can be more beneficial. The interaction between these systems creates a complete modular-based training system which are lacking for engineers currently. Therefore, the goal of this research is to develop a complete modular augmented reality based system that simulates the full planning, control and machining phase for training and education. Finally, the practicability of the developed system needs to be investigated by comparing it to commercially available software tools and through result validation.

To summarise, Table 1 compares the overall features and scope of the proposed work compared to previously developed related research.

## Methods

**Figure 1:**
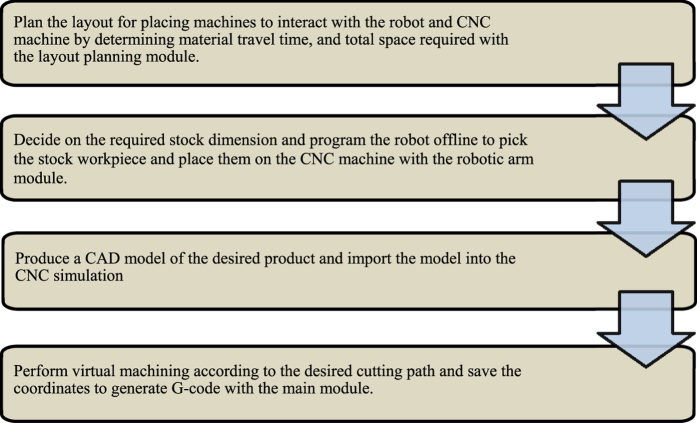
Sequential diagram of the system.

The research is divided into three separate programme modules with its own features which contribute to the overall system, as well as being able to stand alone as an independent system by itself. The planning module focuses on the initial planning stage, which is the application of AR technology in planning the layout of machine placement to optimize the material travel time and area required. The robotic arm module models an AR robot arm with inverse kinematics to pick a workpiece from a conveyor and place it into the 3-axis vertical CNC milling machine. The virtual CNC finally simulates the material removal with collision detection and generates a G-code programme for the actual machining operation. Figure 1 shows the full procedure for an engineer to follow should he or she apply this training system.

**Figure 2:**
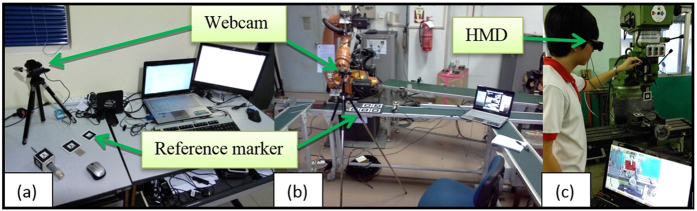
Setup of the system for the (**a**) layout planning, (**b**) robotic arm, and (**c**) CNC module, where the user is viewing the simulation through a HMD.

ARToolKit is used to create a running program that can generate AR content through a marker-based tracking method^46^,^47^. It is essentially a software library for building an AR environment that is rendered with OpenGL. This is the basis of all AR-generated content in this study, where different markers perform different tasks based on the module’s program. Microsoft Visual C++ 2008 express edition is used to compile and debug the C++ codes that will run synonymously with the aid of ARToolKit and OpenGL. On the hardware side, a personal computer is sufficient to run the program and AR environment. Complementing that is a head-mounted-display (HMD) that acts as the display system and a webcam for tracking and registration. The markers, which are symbols and patterns with specific functions which can only be recognised by the AR program, will be placed in its respective working environment. For the layout planning module, a tabletop system is sufficient to calculate the space and time required, but the robotic arm module markers are placed in the robotic lab with an up to scale virtual robotic arm superimposing a physical one for a proper distance estimation. The CNC simulation markers are placed on the CNC machine itself for an *in situ* simulation process. All of these markers must be placed in such a way that it is clearly visible to the camera under direct lighting, and the camera must be placed at a suitable height, as the images of the marker will be blurry if the camera is not adjusted to the correct height. The camera is mounted on an adjustable tripod facing the marker placements, and connected to the laptop. Figure 2 shows the physical setup for each of the module at its respective environment. The individual in this manuscript has given written informed consent (as outlined in PLOS consent form) to publish these case details.

When the camera sees the marker in the real world and is captured in a real time video, the program calls out the specific function associated with the marker pattern. The virtual overlay of the model will appear as though on top of the marker, viewable on the HMD. This means that the virtual content has successfully merged with the real world imagery.

### AR Programming

The fundamental aspect of AR programming is to generate 3D content, which all starts from a single point. This is especially useful in cases where a point such as the robot arm end-effector or the till of the milling bit needs to be visualised. The OpenGL function *glVertex3d* draws a vertex in space where the size and colour can be defined by the user. By creating at least 2 points, a line can then be visualised to connect them. This is used for cases where the path needs to be visualised, such as the material travel path between machines. *GL_LINE_STRIP* draws a line automatically by connecting two vertices together where the line width and colour again is fully adjustable.

Creating full 3D models in the AR environment is achieved by importing a CAD file in stereolithography (STL) and read by the program^48^. STL files actually contain the coordinates of the numerous triangles used to build the model. Hence, the program needs to recognize and read these data. A *ReadSTL* and *DrawSTL* function is created which can recreate the CAD models as fully rendered AR content.

Any form of data acquired from the simulation needs to be exported or generated in some way. Otherwise, it would be difficult to extract live data during the simulation, especially when it is being updated continuously. The program must be able to extract the data from the simulation through a user input and place them into a separate file that can be opened with a text editor. The user input is assigned to a mouse click, where the *GLUT_RIGHT_BUTTON* and *GLUT_DOWN* functions state the condition when the right mouse button is clicked once. This works in a decision making algorithm where it is paired with a *saveCoordinate* function that prints out the coordinate data to a separate output file. All three modules utilise this code function for the user to obtain and edit the parameters obtained in the simulation.

The final set of codes that plays an important role in the entire system is the collision detection algorithm. This refers to the ability to detect objects which are within certain proximity and is achieved by first calculating the relative distance. A more advanced version of the code is applied in the CNC module and will be explained in later sections. Collision detection is extremely important in the manufacturing field because accidents that involve collision between human beings and machines can be fatal. In this system, it is applied to detect collision between machines during layout planning, to recognize the pick-and-place features for the robotic arm, and to simulate material removal. This is achieved with a series of decision making that sets a minimum acceptable distance value and checks if the distance between markers is equal or more than the said value. Otherwise, collision is said to have occurred and the models are rendered red in colour to depict that.

### Work Cell Layout Planning

**Figure 3:**
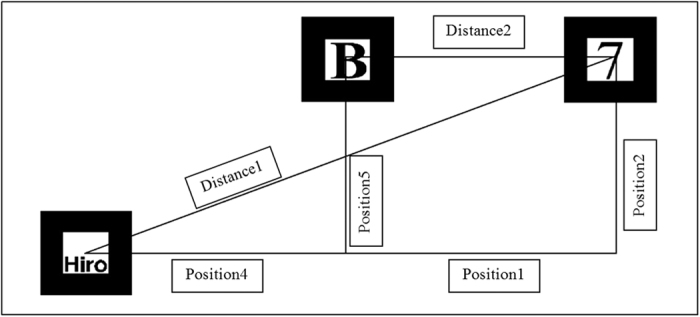
The “Hiro” marker is treated as the world coordinate to calculate the total distance.

The first module, the work cell layout planning, incorporates AR to aid in the development of a flexible manufacturing cell (FMC) by superimposing 3D models of machines into the physical environment while taking into consideration spatial constraints and collision detection^10^. Each of the 3D models present in this module can in fact be scaled to any value. Therefore, the user is free to consider the actual dimension and area required for each machine. However, for ease of analysis and case study, the machines are scaled into a size that is appropriate for a commercial webcam to capture all the markers representing the machines, while still having the models present in the camera’s field of view (FOV). A VR system may also freely scale objects, however, the high computational requirement and lack of spatial awareness of the real environment becomes the limiting factor. Four types of layout are analysed, namely the straight line, U-shaped, S-shaped (serpentine), and semi-circle-shaped environment. A data structure in extensible mark-up language (XML) is then developed to record the information regarding spatial relationship, material travel distance, area occupied, processing time, and sequence of operation. The first marker placed serves as the world coordinate, as well as the first machine in the production line. Every subsequent marker will act as the next machine, and the distance between that marker and the previous one will be the relative distance which exists between each virtual machine, as shown in Fig. 3.

**Figure 4:**
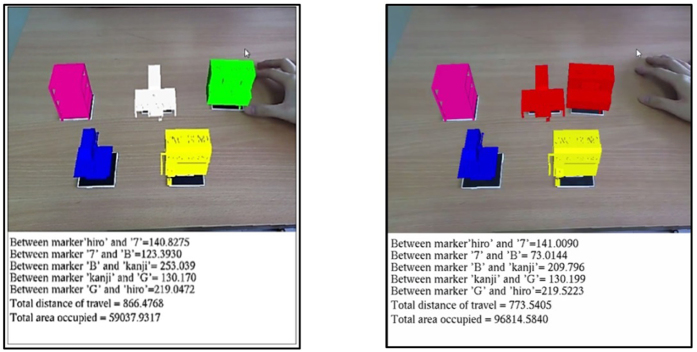
Colour change of the machine to indicate collision in the running layout program.

Since the area required is also taken into consideration, the program must be able to identify which marker has the longest distance relative to the reference marker. By doing so, the system knows the largest possible area that the work cell will require; assuming the space given is rectangular or square in shape. The integrated collision detection code causes a change in colour of the virtual machines when collision is registered as shown in Fig. 4, letting the user know that the machines are placed too closely together.

The determination of the best layout will be conducted at the case study section, where the four different types of layout mentioned previously are tested to determine its effect on the cycle time in line balancing. Line balancing is the key method in designing the most efficient process that is in line with the expected volume or demand of a product, but it rarely takes into account the time required for materials to move between stages and how this affects the cycle time which focuses on processing time^49^. In fact, line balancing is computed by finding the required number of stages based on the cycle time, which by definition is the time taken for a product to emerge from a stage. Cycle time, *t*_1_ is computed based on the available time, *t*_*a*_ and the demand, *d*. The value of *t*_*a*_ and *d* is determined by the user based on their specific requirements. Next, a product requires several operations to manufacture or assemble, and each operation has its specific time, *t*_*operation*_ required to complete it depending on the operation’s complexity and requirements. The total number of stages, *S* can be found by dividing the total work content, *T*_*n*_ with *t*_1_. Each machine in the AR environment is treated as a single stage, where the system then computes the material travel distance with the travel speed is decided by the user. Therefore, the material travel time, which is the time taken for a material to move from the first stage to the last stage, *t*_m_ can be found. *t*_m_ can then be added back into *T*_*n*_ to obtain the total operation time. The new cycle time, *t*_2_ which is computed by considering the material travel time represents a more accurate cycle time as it sums travel time with the actual work content. This allows engineers to carefully consider which layout arrangement is most suited for their required operations because the inclusion of standard time for material flow reduces the risk of late delivery of the final product^50^. The full formulation is shown below.

If


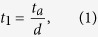


Then


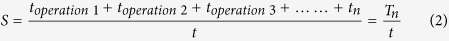


With the addition of *t*_m_, New cycle time,


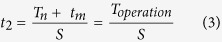


### Robotic Arm Pick-and-Place Operation

**Figure 5:**
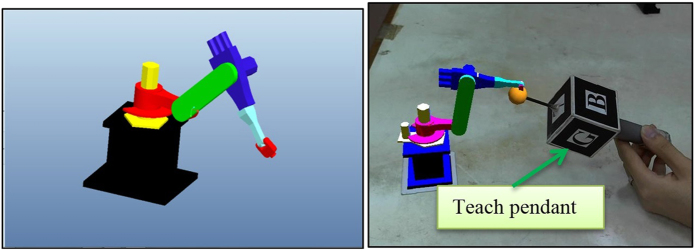
3D CAD model of the robot arm imported into the virtual environment, where the end effector follows the teach pendant.

In a pick-and-place operation, the arm does not perform a specific operation like welding, soldering, and so on. Therefore, the focus on the kinematic study of the robot is important to obtain accurate modelling when creating a virtual robot arm^51^. This module emphasises the robot’s kinematic study based on the KUKA KR 16 KS robot as well as the functions used to obtain a snapping visualisation to pick and place a virtual workpiece. Pro-Engineer is used to model the robot to scale and joint by joint, then assembled together in OpenGL to create a full virtual robot arm as shown in Fig. 5 where each joint can be manipulated at a variable angle.

**Figure 6:**
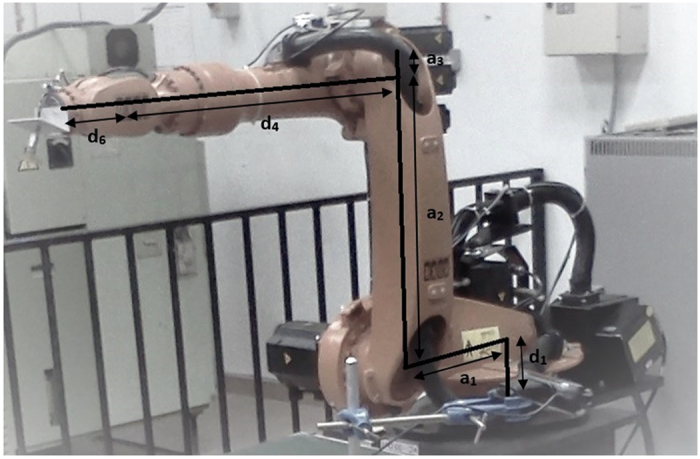
Link length and link offset of the KUKA KR 16 KS robot arm.

According to D-H kinematics, each compartment of the robot is first assigned a coordinate frame with the origin assigned to the top surface of the pedestal. The primary goal is to obtain the angle of each joint which results in the end effector position. These angles can then be used on the physical robot arm programming. A D-H coordinate frame consists of four parameters, *a*, *α*, *θ*, *d* which are the link length, link twist, joint angle, and link offset respectively. The linkage is illustrated in Fig. 6, while Table 2 demonstrates how the parameters are linked. The general equation for forward kinematics is the product of the matric transformations from frame 0 to frame 7.


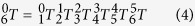


where each value of 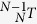 represents each joint.

**Figure 7:**
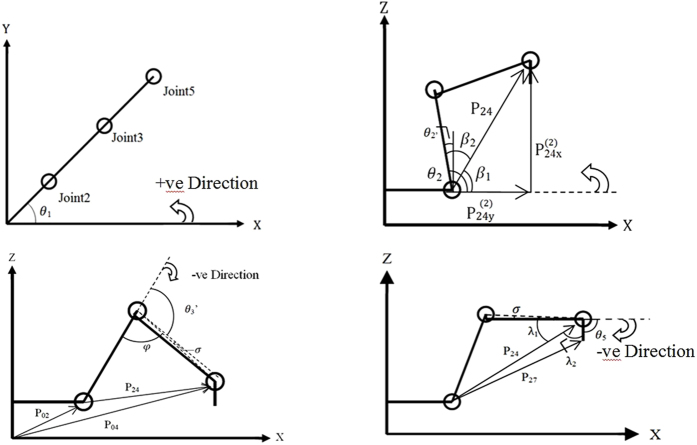
Free body diagram to compute *θ*_1_, *θ*_2_, *θ*_3_, and *θ*_5_ respectively^51^.

This gives us the formulation for the forward kinematics as well as the end effector position. *P*_*x*_, *P*_*y*_ and *P*_*z*_ represents the end effector coordinates.


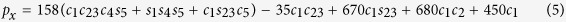



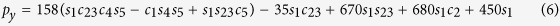



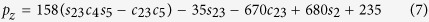


where *c*_*n*_ and *s*_*n*_ represents cosine and sine for the respective matrices. However, inverse kinematics is required to obtain the joint angle of the arms. Once the angle of each arm is determined, the robot can then use these values to obtain the desired end effector coordinate. A limitation is placed on *θ*_4_ and *θ*_6_ to reduce the probability for an error to take place, since these joints are twist joints which should not affect the consecutive joint’s coordinate, and that the end effector will simply face downwards. Figure 7 shows the free body diagram of the other joints, where *θ*_1_ is shown in the X-Y plane rotating about the Z-axis, and *θ*_2_, *θ*_3_ and *θ*_5_ are shown in the X-Z plane rotation about the Y-axis. This method of computing the angles are detailed in a recent paper that explains the joint assumptions that were made^51^.

**Figure 8:**
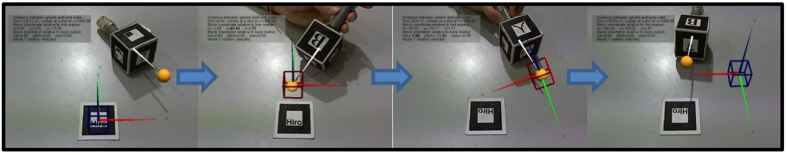
Pick and place sequence of the virtual object. The blue wireframe cube that was picked turns red when in contact with the teach pendant, and returns blue once placed at another position.

**Figure 9:**
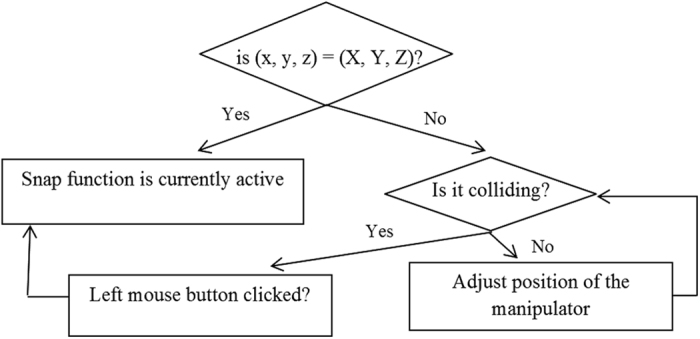
Snapping function algorithm.

Once the kinematic modelling of the robot is completed, the pick and place operation is initiated. The teach pendant must be able to manipulate the virtual stock in space, to show that the robot arm is picking and placing the stock around. This is called snapping, where an object immediately takes a position in space when an operation is performed. With a single mouse click, the virtual object takes the position of the tip of the manipulator with the condition that it is colliding. The user can also choose to drop the object anywhere in space simply by releasing the mouse button since the program is designed to continuously update the most recent position of the virtual object. (x, y, z) refers to the current position of the virtual object, while (X, Y, Z) refers to the position of the teach pendant tip at the moment the mouse button is clicked. The resulting effect is shown in Fig. 8, whereas the function algorithm is shown in Fig. 9.

### CNC Machining Simulation

The main purpose of the final code module is to machine out the final product based on the design of the user. To achieve this, a modified collision detection system, machining parameters, heads-up-display (HUD) and G-code generation will be integrated together.

The previously utilised collision detection algorithm simply calculated the distance between two points based on the formulation stated below.


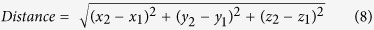


**Figure 10:**
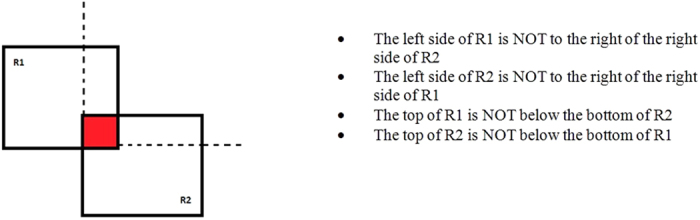
AABB during intersection.

However, this formula implies that both of the points are considered as the centre point of a sphere-shaped object since the distance between them is constant. If a maximum allowable distance was set, such as a value of 100 cm, this will be equivalent to two spherical object of radius 50 cm touching each other at a single point. Therefore, this algorithm can only be applied to find collision between two points or spheres with no edges or corners present. In this study, it is assumed that the stock workpiece is a single block of material and the cutter is bounded by a rectangular box, which therefore requires a collision algorithm suitable for cuboid objects. Furthermore, the nature of vertical milling requires a variable depth from the top surface of the workpiece to visualize the depth of cutter engagement. The axis-aligned bounding box (AABB) algorithm is used to fulfil these requirements. As a bounding box or a typical 2D box is made of four sides, the routine requires four conditions which are the four corners. The intersection method is based on the simple logic in Fig. 10.

**Figure 11:**
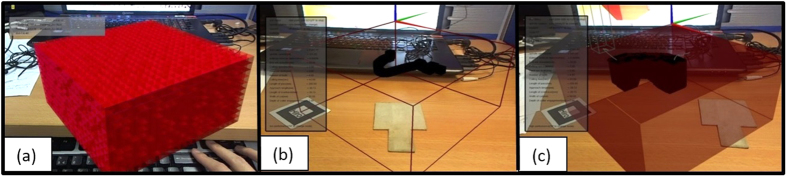
(**a**) USD stock rendering, (**b**) stock in wireframe, and (**c**) blackened cubes to indicate collision.

To apply this logic into the simulation, the boxes must first be transformed into 3D cubes. Both the stock and cutter will be treated as a bounding box. However, an inaccurate visualization will occur if the typical AABB method is used. If the entire cutter is placed into the workpiece, AABB collision will cause the visualization of only the intersection between the two boxes, which means a floating black box inside the workpiece. In an actual milling operation, this will result in a depth of the cut from the surface of the workpiece until the tip of the cutter, assuming that an operator actually cuts into a material until the depth of cutter engagement is higher than the actual length of the cutter. In other words, the depth of cut needs to be set as a variable, unlike the width and length. This is reflected in the code where the z-axis value only evaluates the lower surface and not the top of the cutter. Instead, the top is associated with the top surface of the workpiece instead. A variable *dcut* is defined as the depth of cut, or the difference in height between the top surface of the workpiece and the lower surface of the cutting tool. The uniform space decomposition (USD) method is also used to represent the stock workpiece so that it can be visualised as material being cut. In a USD-based method, the stock workpiece is represented as cubes, spheres, or any shape of the same size^52^. This means that the entire stock is made up of smaller cubes, where the size of each cube determines the resolution of the object. When the cutter or tool passes through the cubes, cubes which intersect with the tool during the process are rendered black, and eventually the volume of the black cubes represents the outcome of the machining process. Figure 11 shows the USD-based stock, together with the visualisation of depth of cut.

The parameters involved in the simulation aids the user in understanding its effect through real-time visualisation depending on the current operation. These parameters are divided into the user input and the calculated output. Unlike the robot arm, kinematic modelling of the actual CNC machine is not included in this code module and thus, the axis movement and trajectory planning are borrowed from the physical machine by placing the markers directly on the machine itself. However, not all CNC machines calculate the machining parameters for the user, and thus are included in this program.

N  =  RPM of Cutter, or Spindle Speed

n  =  Number of Teeth on Cutter

W = Width of cut (may be full cutter or partial cutter)

T  = depth of cutter engagement

V  = cutting speed (Handbook value)

L  = Length of pass or cut

f_m_  = Table (machine) Feed, or Feedrate

f_t_  = feed per tooth of cutter, or Chip Load (Handbook value)

D  = Cutter Diameter

L_A_  = Approach Length

L_O_  = Length of “OverTravel”, where the turret moves beyond its boundaries

Then,

Spindle speed,


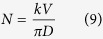



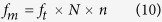


Let


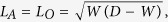


Then, Cutting time,


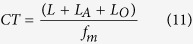



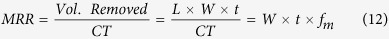


**Figure 12:**
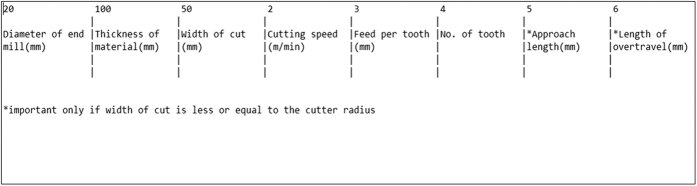
The “machining_parameter.dta” file which details the entire user input data.

The *sscanf* function in the code allows the system to read values from an external file in the same directory in the program. For the user to enter the necessary parameters, a separate file called *machining_parameters.dta* is included where he or she simply needs to input the first initial six values which are the cutter diameter, workpiece thickness, width of cut, cutting speed, feed per tooth, and number of tooth, to compute all the necessary parameters. Figure 12 shows the file with a detailed explanation of each parameter and what they represent. Therefore, the user does not need to constantly input the values each time and just change them in the separate file should the need arise.

**Figure 13:**
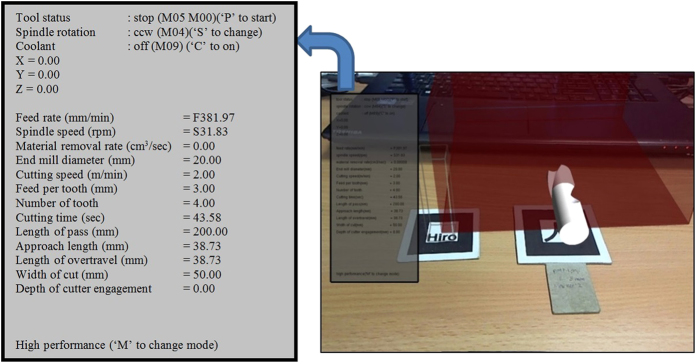
A HUD at the left side of the screen with information overlay.

The addition of a HUD is extremely useful when virtual content is involved in any context. It extends our knowledge of the current operation when it is performed, and continuously updates itself with the current situation as well. The information overlay covers the current tool state with related G-code, spindle rotation direction, coolant condition, and all of the aforementioned parameters. The live update feature ensures key information like current tool coordinate, MRR, and cutter depth engagement constantly changes to reflect the current machining conditions. To achieve this, the perspective of the model needs to negate the initial global origin which will cause the HUD to move around if the “Hiro” marker is moved. Additionally, a semi-transparent background is used to increase the visibility of the words without obscuring the operation much. The following functions were utilised:

glMatrixMode (GL_PROJECTION);

glMatrixMode (GL_MODELVIEW);

glEnable (GL_BLEND);

glBlendFunc (GL_SRC_ALPHA, GL_ONE_MINUS_SRC_ALPHA);

Lastly, the *printw* function prints all the relevant information into the AR scene. The resulting effect is as shown in Fig. 13.

With the models accurately visualised, the system needs to generate G-codes which can then be used for the actual CNC machining. One of the key features of the simulation system is the ability to generate G-code blocks based on the virtual environment and the placement of the cutting tool relative to the workpiece coordinate system (WCS). Despite the system supporting 3-axis only, complex operations can still be carried out, evident by past applications even for non-uniform surfaces like sculpturing with NC machining^53^. Furthermore, extension to 4- and 5-axis CNC machines can be done once 3-axis machining is properly established^54^. The list of supported G-codes is shown in Table 3.

**Figure 14:**
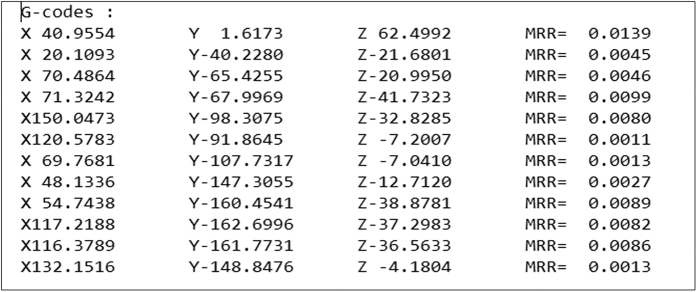
Saved output text file.

These codes can all be seen on the HUD with visual cues, such as the workpiece becoming blue in colour when the coolant is switched on. The key values in a G-code programming, which are the X, Y, and Z values are tied to the *saveCoordinate* function which is specifically designed to operate with the mouse input to save the current coordinate when the mouse button is clicked. An example of the text file is shown in Fig. 14 which is generated based on a total of 12 mouse clicks.

### Case Study

The case studies are designed as a form of validation process to observe how much the parameters deviate over conventional tools and to reflect the error present in the system^26^,^29^,^48^,^55^,^56^,^57^. This is to prove that the developed system has the potential to replace them, with the added benefit of it being more immersive, realistic, having a better sense of depth, with real-time information feedback, and a better simulation experience overall. Since the modularity of this system is emphasised, the case study is conducted in a fashion where each module is treated as a standalone system. Validation software tools like Mastercam and Kuka Sim Pro play a major role as they are primarily needed to validate the results generated by the AR environment.

### Layout Planning Case Study

**Figure 15:**
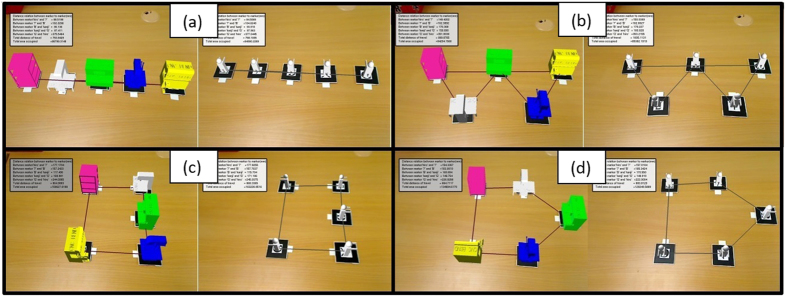
Virtual layout of machine and operators with various arrangements, with (**a**) straight line arrangement, (**b**) S-shaped arrangement, (**c**) U-shaped arrangement, and (**d**) semi-circle-shaped environment.

A case study is conducted based on the manufacture and assembly of a computer case. For ease of calculation, input values are kept at the lower range to reduce the computed value of the number of stages, since it can range from 0 to 100 stages in an actual production line. For this particular case study, *t*_*a*_ = 8 hours/day, *d* = 800 units/day and *n* = 6 operations. Since each operation has its own required time, *t*_*operation*1_ = 45 seconds, *t*_*operation*2_ = 18 seconds, *t*_*operation*3_ = 22 seconds, *t*_*operation*4_ = 32 seconds, *t*_*operation*5_ = 20 seconds and *t*_*operation*6_ = 43 seconds. The program uses these values to find *t*_1_ and *S*, which is equal to 36 seconds and 5 stages respectively. The goal of this case study is to acquire the best possible route with minimum material flow distance and the least space required. Several line shapes were evaluated, that includes the straight –line, S-shaped, U-shaped, and semi-circle-shaped as shown in Fig. 15. Furthermore, two forms of orientation which represents an automated and a manual line, which are machine-centred and operator-centred respectively, are both analysed.

Based on the result in Table 4, the lowest value of the total distance travelled is at 328.52 m, which is the operator-oriented method with a semi-circle-shaped arrangement. Additionally, this particular arrangement also scores the least distance travelled for a machine-centred oriented operation. Assuming the speed to be 0.33 m/second, this equals to a travel time of 108.4 seconds. We can then find *t*_*2*_, according to equation (3).


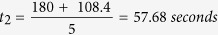


### Robotic Arm Case Study

**Figure 16:**
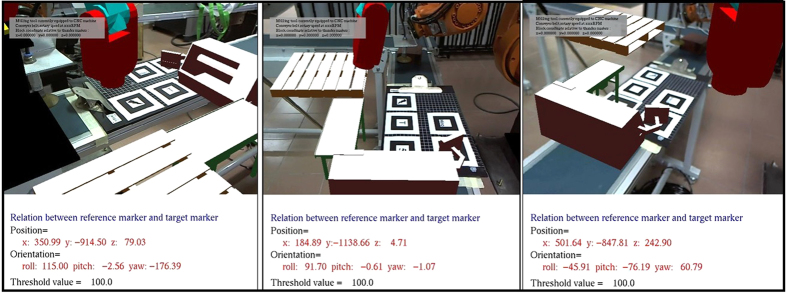
The virtual robotic arm module at full scale viewed at different angles through a HMD as the user walks around the test area.

Validation of the developed module is conducted by utilising the virtual machines from the layout planning module to create a robotic work cell. The operation involves the picking and placing of a block of material around the various virtual machines. The user guides the end effector of the virtual robot arm by manually pointing at the locations for picking and placing using the teach pendant. The coordinate and angle of the arm at that point is then saved into a separate file. The saved angle values are input into Kuka Sim Pro, where each of the points can be compared and validated. Figure 16 shows multiple views of the work cell used for the case study.

Kuka Sim Pro is an offline programming software that fully simulates the activities of the robot arm, and therefore is no different from the actual coordinate inputted into the teach pendant of the physical robot. The generated angles of each arm from the AR environment are input into Kuka Sim Pro to generate the corresponding coordinates of the end effector. The results are shown in Table 5, where the simulation is validated with a maximum error of 3.83 mm.

### CNC Machining Case Study

**Figure 17:**
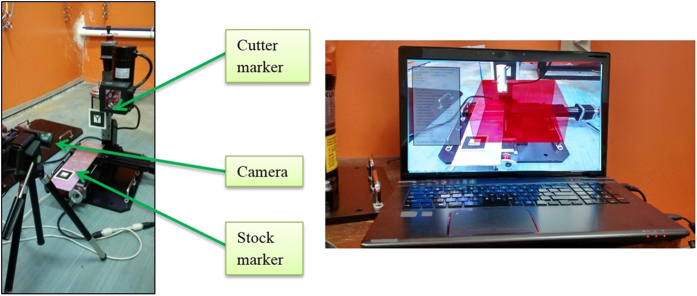
Setup for testing the simulation on a table-top CNC machine.

The machining operation will be conducted in a fashion similar to Mastercam, as well as validated with Mastercam to determine the accuracy of the operation. A CAD model was created using Pro-Engineer with the overall dimensions being the same as the workpiece in the simulation. The imported 3D model carries over the dimensions of the CAD model with the same machined slots, as an overlay on top of the original virtual stock. This is essentially how Mastercam works, however Mastercam does not provide an *in situ* simulation system. The generated G-code is then compared with the Mastercam G-code and any inconsistencies were observed. Figure 17 shows how the simulation process was carried out on a physical milling machine to ensure an accurate axis movement of the cutter.

**Figure 18:**
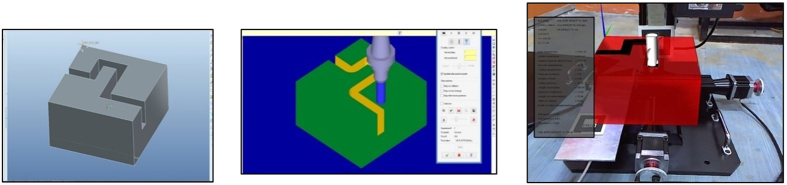
Imported CAD model, Mastercam simulation, and AR simulation.

The case studies will be separated to test the machining capabilities of each axis individually. The designed stock size is 200 mm × 200 mm × 100 mm, as well as the dimensions of the virtual stock in the AR system. The cutter marker can roughly be placed on the spindle area of the physical cutter as long as it remains sturdy. Similarly, the stock marker does not need to be placed on the actual vice with the mounting coordinates known. This is because its placement will not affect the generated G-code, as the values are calculated relative to the virtual workpiece and the virtual cutter. Conducting the case study on a physical milling machine is merely to provide an accurate axis movement for the cutter while at the same time, providing an actual machining or manufacturing environment to the operator. Figure 18 shows the machining simulation for cutting about the x and y-axis.

**Figure 19:**
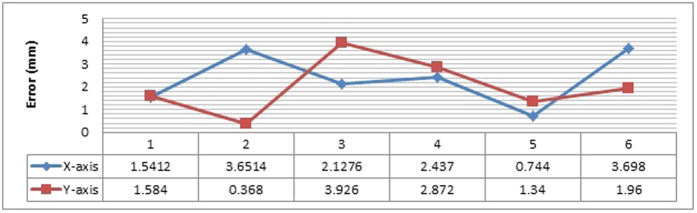
Error graph for the X-Y cutting validation.

From the G-codes generated through Mastercam, the main machining values are then extracted and compared side by side between both the simulation systems. The graph shown in Fig. 19 illustrates the error present in both the axes on its deviation from the Mastercam results, which is assumed to be ideal and error-free.

**Figure 20:**
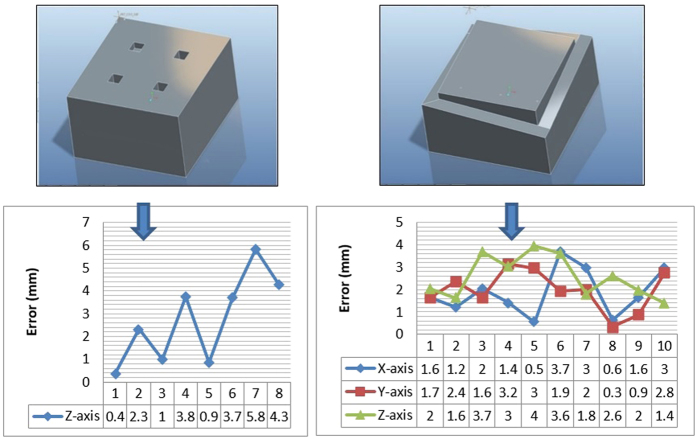
CAD model of the stock to generate the error graphs for Z-axis and 3-axis validation.

The same procedure is used to find the error present when machining about the z-axis and finally complex machining is performed for all three axes, as shown in Fig. 20. The z-axis machining case study drills four holes with different depths at an increment of 10 mm. The 3-axis machining case study is carried out because typically, a finished product requires machining of at least all three of the axes, therefore this validation is the most accurate representation simulated of a real product. The cutting is performed on the edges with a slope-like design for a variable depth.

It is observed that the highest deviation from the Mastercam software for all the case studies is point 7 for the z-axis machining, with a value of 5.8 mm which is considerably higher when compared to the x-axis, y-axis, or 3-axis machining.

## Discussion

For the layout planning module, 57.68 seconds is a more accurate representation of cycle time using the semi-circle shaped arrangement. For the total area required, the straight line layout requires the least area, at 19833.14 m^2^. A smaller space would obviously be favourable; however, this depends on additional factors like the amount of allocated space in the first place, as well as the space required for other work cells. As for the robotic arm module and the CNC machining module, the highest deviation errors produced were 3.83 mm and 5.8 mm respectively. It needs to be understood that in both cases, the presence of these errors is unavoidable even in commercially available software programmed by first party developers. Simulation will always differ slightly with its physical counterpart; therefore, a certain error value needs to be decided upon as acceptable in this new form of simulation. Judging by the accuracy of ARToolKit itself and coupling it with the implemented algorithms, these errors were deemed acceptable at this point of time. The formulas for the transformation and rotation matrices are correct, because there would be a large deviation among the results if the supporting formulas are wrong. Additionally, with regards to the robotic arm module, hand jitter by the user also plays a role because the human hand is never 100% static^58^.

**Figure 21:**
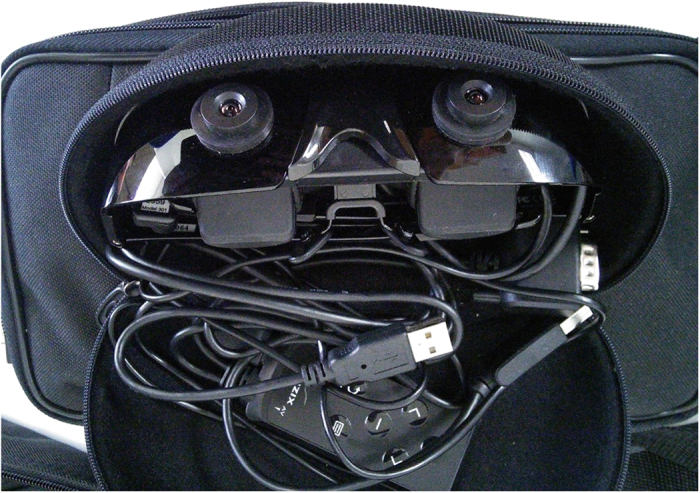
The HMD utilised for this work, with video see-through capabilities.

Most of the illustrated figures are shown as being conducted with a simple computer monitor displaying the AR content. This is so that each generated model can be clearly illustrated on the monitor. However, using a HMD is highly recommended, as shown in Figs 2(c) and 16. This provides the user with an immersive view of the AR content superimposing his or her FOV. The exact HMD model used is the Vuzix Wrap 920AR which is shown in Fig. 21 that provides a video see-through vision with built-in cameras and display. This essentially means that the HMD replaces both the camera and computer monitor for the user. While it is not a requirement, the usage of a HMD can greatly benefit the *in situ* simulation aspect of AR, where it is only natural and intuitive to look at the machine when performing the simulation, as opposed to referring to an external monitor. Tasks like pick-and-place and machining also requires viewing from different angles to assess the situation carefully, which is a benefit from utilising a HMD.

Integration of all these modules as a complete system merely involves the steps illustrated in Fig. 1. As of this moment, the case studies were meant to showcase the ability of each module to act as a standalone system. This freedom that is provided to the user is an important factor in this study, so that using them sequentially is always an option, as for using them as a standalone system or in any other sequences. Otherwise, inclusion of additional dependencies that ties these modules together in a program will become a limiting factor instead, and goes against its modular nature. A separate work is possibly required for this task.

### Conclusion and Future Works

In this paper, a virtual modular-based automated factory operation system for teaching and learning has been developed. This is especially useful to bridge the gap between simulation and actual factory operations, as one of the key benefits of AR is superimposing the actual environment for a better sense of placement. The modular nature of the system allows the scope to be further expanded through additional modules where each plays a part for a complete training system that facilitates automatic machining. Modules like the layout planning and pick-and-place operation teaches the user about the effects of proper machine placement and robot programming respectively, and how it contributes to the production of an end product from the start to finish. Finally, with any simulation system, the developed AR system aims to eventually reduce the lead time and increase the productivity by being more intuitive and immersive.

This study can be further improved by including a graphical user interface (GUI) which is an improvement over the HUD implementation. Further studies should also be conducted to identify and investigate the errors in the results^59^ which can be due to several factors such as, lighting conditions, marker design and hand jitter. Currently, the errors present are still acceptable in the methodology used for validation or learning purposes, but they most definitely need to be addressed to increase user acceptance. Moreover, with regards to the CNC system, circular interpolation was not supported and the AABB algorithm has caused the visualisation of material removal to be only rectangular in shape, which can be both further improved. Finally, a tighter integration while maintaining the modular characteristics of the system would be a huge improvement, such as linking the layout planning module directly to the robotic arm and CNC machining simulation for a better material flow. With these improvements, previously developed methodologies for validation^60^ can be used to determine the simulation accuracy.

In terms of measuring immersion, a separate study is required to determine the level of which immersion can be quantified. It was stated that this depends on many factors like FOV, field of regard, display size and resolution, stereoscopy, head-based rendering, realism of lighting, frame rate, refresh rate, and so on^1^. It is best to acquire other forms of AR systems so a direct comparison can be made for the level of immersion each provides.

## Additional Information

**How to cite this article**: Pai, Y. S. *et al.* Virtual Planning, Control, and Machining for a Modular-Based Automated Factory Operation in an Augmented Reality Environment. *Sci. Rep.* **6**, 27380; doi: 10.1038/srep27380 (2016).

## Figures and Tables

**Table 1:** Comparison table between other related works.

	VR simulation^2^	ARCNC^3^	Virtual factory layout^6^	Mixed reality layout planning^7^	VR-Rocell^9^	AR Robot Programm-ing^21^	The authors’ research
Technology Used	virtual reality	augmented reality	virtual reality	virtual and augmented reality	virtual reality	augmented reality	augmented reality
Scope	vertical milling only	vertical milling only	layout planning only	layout planning and robot arm interaction	robot arm interaction	robot arm interaction	layout planning, vertical milling, robot arm interaction
G-Code	–	acts as input	–	–	–	–	Outputs G-code
CAD Format	VRML	–	DXF	Delmia software	STL	–	STL
Axis/DOF	3-axis	3-axis	–	6-DOF robot arm	6-DOF robot arm	n-DOF	3-axis machining, 6-DOF robot arm
Material Removal Visualisation	none due to high computational requirement	supports real time visualisation	–	–	–	–	supports real time visualization
Layout Planning Method	–	–	finds shortest material travel distance	Delmia based layout-planning	–	–	finds best formation and least area
Robot operation	–	–	–	Delmia-based simulation	pick-and-place	CFV for path planning	pick-and-place

**Table 2:** Denavit-Hartenberg parameters.

Joint *i*	Rotation *α*_(*i*−1)_	Link Length *a*_(*i*−1)_	Joint angle *θ*_*i*_	Link offset *d*_*i*_
1	0	0	*θ*_1_	*d*_1_ = 235 *mm*
2	90°	*a*_1_ = 450 *mm*	*θ*_2_ = *θ*_2′_ + 90°	0
3	0	*a*_2_ = 680 *mm*	*θ*_3_ = *θ*_3_ + 90°	0
4	90°	*a*_3_ = 35 *mm*	*θ*_4_ = 0	*d*_4_ = 670 *mm*
5	−90°	0	*θ*_5_	0
6	90°	0	*θ*_6_ = 0	*d*_6_ = 158 *mm*

**Table 3:** List of supported G-codes.

G-Code	Description
G00	Rapid Linear Interpolation
G01	Linear Interpolation
G21	Machine in mm
G90	Absolute command
M-Code	Description
M00	Program stop
M03	Spindle On Clockwise
M04	Spindle On Counter clockwise
M05	Stop spindle from turning
M08	Coolant On
M09	Coolant Off
Other Codes	Description
F	Feed rate/Dwell time in seconds
S	Spindle speed
X	Code for the X-axis
Y	Code for the Y-axis
Z	Code for the Z-axis

**Table 4:** Total travel distance and area for each layout.

Layout Arrangement	Total Travel Distance(m)		Total Area (m^2^)
	Machine centre	Operator	
Straight	662.61	671.74	19833.14
S-Shaped	696.79	494.58	43655.28
U-Shaped	553.71	432.40	34225.45
Semi-circle Shaped	476.26	328.52	31472.27

**Table 5:**
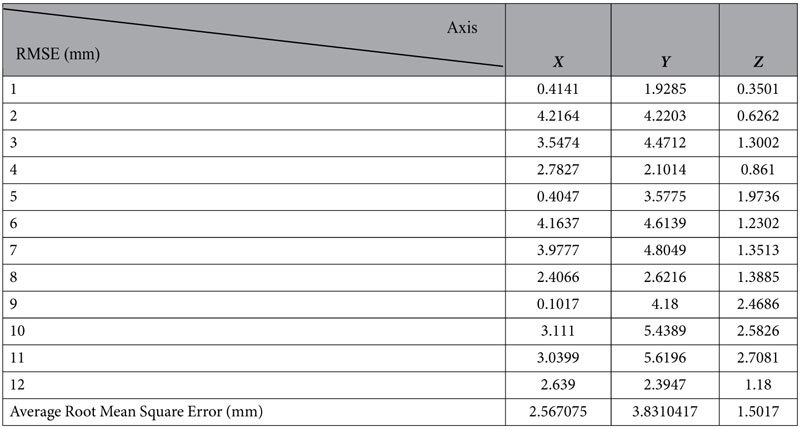
Calculated root mean square error (RMSE) value.
