# Erratum: Virtual Planning, Control, and Machining for a Modular-Based Automated Factory Operation in an Augmented Reality Environment

**DOI:** 10.1038/srep31678

**Published:** 2016-08-25

**Authors:** Yun Suen Pai, Hwa Jen Yap, Siti Zawiah Md Dawal, S. Ramesh, Sin Ye Phoon

Scientific Reports 6: Article number: 27380; 10.1038/srep27380published online: 06 07 2016; updated: 08 25 2016

In this Article, Hwa Jen Yap, Siti Zawiah Md Dawal, S. Ramesh and Sin Ye Phoon are incorrectly affiliated to ‘Graduate School of Media Design, Keio University, 4-1-1, Hiyoshi, Kohoku, Yokohama, 223-8526, Japan’. The correct affiliation is listed below:

Department of Mechanical Engineering, Faculty of Engineering, University of Malaya, 50603, Kuala Lumpur, Malaysia.

